# Loss of PPARγ expression by fibroblasts enhances dermal wound closure

**DOI:** 10.1186/1755-1536-5-5

**Published:** 2012-04-13

**Authors:** Wei Sha, Katherine Thompson, Jennifer South, Murray Baron, Andrew Leask

**Affiliations:** 1 Department of Dentistry, Schulich School of Medicine and Dentistry, Western University, Dental Sciences Bldg., London, ON, N6A 5C1, Canada; 2 Department of Rheumatology, Jewish General Hospital, 3755 Cote St. Catherine Rd., Montreal, QC, H3T 1E2, Canada; 3 Department of Physiology and Pharmacology, Schulich School of Medicine and Dentistry, Western University, Dental Sciences Bldg., London, ON, N6A 5C1, Canada

## Abstract

**Background:**

Peroxisome proliferator-activated receptor (PPAR)γ may be a key regulator of connective tissue deposition and remodeling *in vivo*. PPARγ expression is reduced in dermal fibroblasts isolated from fibrotic areas of scleroderma patients; PPARγ agonists suppress the persistent fibrotic phenotype of this cell type. Previously, we showed that loss of PPARγ expression in fibroblasts resulted in enhanced bleomycin-induced skin fibrosis. However, whether loss of PPARγ expression in skin fibroblasts affects cutaneous tissue repair or homeostasis is unknown.

**Results:**

Mice deleted for PPARγ in skin fibroblasts show an enhanced rate of dermal wound closure, concomitant with elevated phosphorylation of Smad3, Akt and ERK, and increased expression of proliferating cell nuclear antigen (PCNA), collagen, α-smooth muscle actin (α-SMA) and CCN2. Conversely, dermal homeostasis was not appreciably affected by loss of PPARγ expression.

**Conclusion:**

PPARγ expression by fibroblasts suppresses cutaneous tissue repair. In the future, direct PPARγ antagonists and agonists might be of clinical benefit in controlling chronic wounds or scarring, respectively.

## Background

If the dermis is injured, specialized fibroblasts called myofibroblasts repopulate the wound and synthesize and remodel new connective tissue [1]. Wound repair is very complex and dynamic, involving the interactions of multiple cell types and growth factors/cytokines; dysregulation of this process results in chronic wounds or fibrosis [2]. Thus, understanding the molecular mechanisms controlling the normal tissue repair program is likely to be of clinical relevance.

Expression of the nuclear receptor peroxisome proliferator-activated receptor (PPAR)-γ is reduced in dermal fibroblasts isolated from fibrotic lesions of patients with the autoimmune connective tissue disease scleroderma (systemic sclerosis, SSc); moreover, the PPARγ agonist rosiglitazone reverses the persistent fibrotic phenotype of this cell type [3]. Normally, PPAR-γ is bound to the retinoid X receptor (RXR) and co-repressors, preventing its binding to DNA; however, upon receptor ligation, the co-repressors are displaced from the PPAR-γ/RXR complex allowing PPAR-γ to bind PPAR-γ response elements in the promoters of target genes [4]. The PPAR/RXR transcriptional complex plays a critical role in maintaining energy balance, which is dysregulated in conditions such as obesity, diabetes, and atherosclerosis [4].

An increasing body of evidence also suggests that PPAR-γ plays a key role in connective tissue turnover, a key process involved with tissue remodeling [5]. Both *in vivo *and *in vitro*, PPAR-γ agonists inhibit basal and transforming growth factor (TGF)-β-induced collagen deposition and myofibroblast differentiation [6,7]. Although loss of PPARγ expression in cultured mouse embryonic fibroblasts results in enhanced, constitutive Smad3 phosphorylation and collagen production [8], loss of PPARγ expression in cultured adult mouse fibroblasts appears to be insufficient to result in either Smad 3 activation or collagen production [9]. Instead, adult dermal fibroblasts lacking PPARγ expression show enhanced sensitivity to exogenously added TGFβ in terms of enhanced phosphorylation of Smad3 and expression of collagen/α-SMA mRNA [9]. Collectively, these data strongly suggest that PPARγ may play a role in fibrosis by enhancing cellular responses to TGFβ.

Recently, we showed that mice harboring a fibroblast-specific deletion for PPARγ displayed an enhanced susceptibility to bleomycin-induced skin scleroderma [9]. These observations suggest that PPARγ might be an important regulator of cutaneous tissue repair and homeostasis *in vivo*; however, this hypothesis has yet to be tested. Herein, we subject mice harboring a fibroblast-specific deletion of PPARγ to the dermal punch model of cutaneous tissue repair. Moreover, we assess whether loss of PPARγ expression by skin fibroblasts affects dermal homeostasis. Our results reveal new insights into the role PPARγ plays in fibroblast biology.

## Results

### Loss of PPARγ expression in skin results in faster wound closure

**Figure 1:**
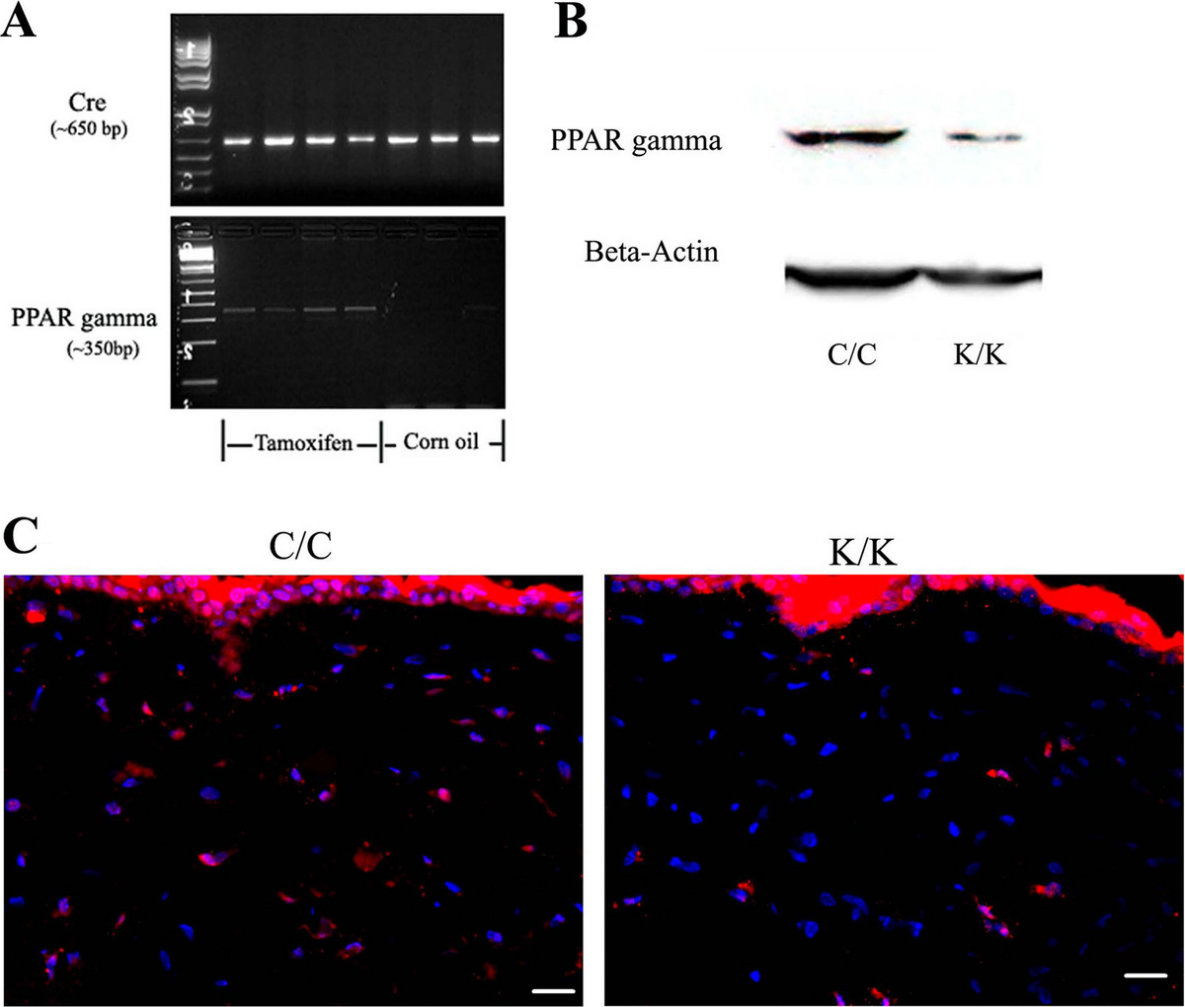
**The deletion of PPARγ in skin fibroblasts**. **(A) **Mice homozygous for loxP-PPARγ and hemizygous for an allele enabling a tamoxifen-dependent cre recombinase to be expressed under the control of a fibroblast-specific collagen type I promoter/enhancer were injected with tamoxifen or corn oil to generate mice deleted (K/K) or not (C/C) for PPARγ in fibroblasts. PCR genotyping of PPARγ WT (C/C) or PPARγ KO (K/K) mice. The upper panel shows the PCR result using primers detecting cre (approximately 650 bp band); the bottom panel shows the PCR result with specific primers which amplify Exon I and Exon II-deleted PPARγ after tamoxifen injection (approximately 350 bp band). **(B) **Western blot analysis of WT (C/C) and PPARγ KO (K/K) fibroblasts with an anti-PPARγ antibody. **(C) **Indirect immunofluorescence analysis of WT (C/C) and PPARγ KO (K/K) mice skin samples (original magnification × 40, bar = 25 μm) using an anti- PPARγ antibody. Red = PPAR gamma; Blue = DAPI. DAPI, 4',6-diamidino-2-phenylindole; PPARγ, peroxisome proliferator-activated receptor-γ.

**Figure 2:**
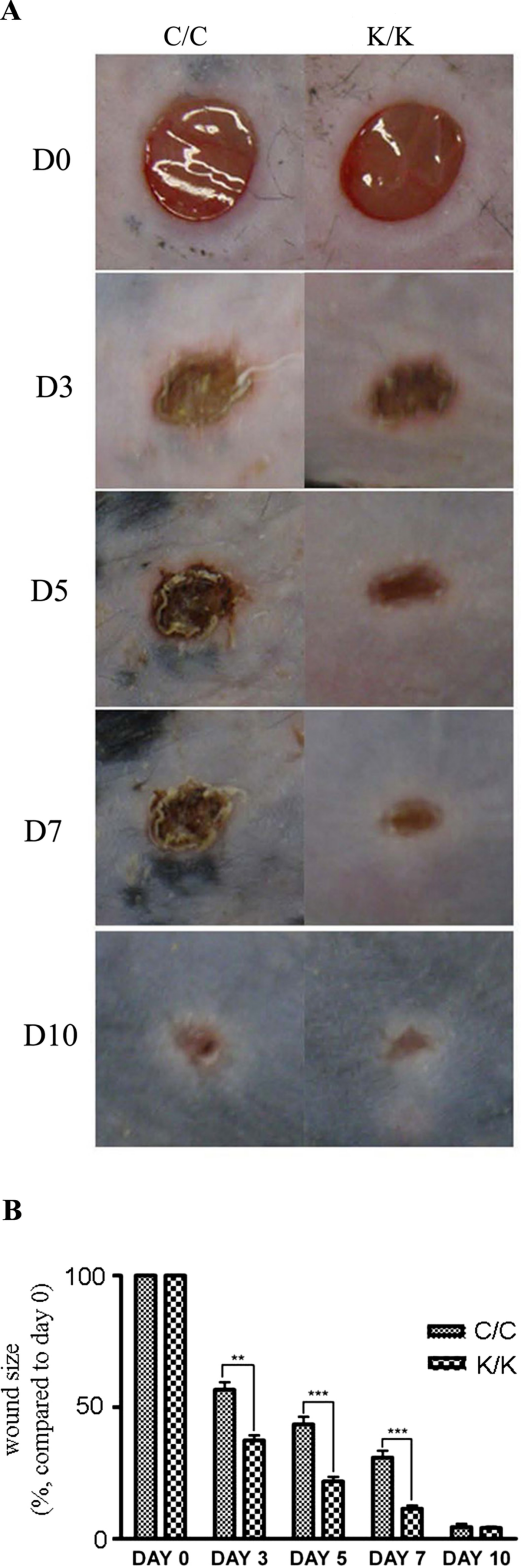
**Wound closure is faster in PPAR**γ **KO (K/K) mice than WT (C/C) mice**. **(A) **Photography of wound sites at different time points after wounding (N ≥ 12 for each time point, four wounds for each mouse) **(B) **Quantification of wound size at different time points after wounding. Asterisks indicate significant differences between WT and KO groups (** = *P *< 0.01; *** = *P *< 0.001). D, day. PPARγ, peroxisome proliferator-activated receptor-γ.

**Figure 3:**
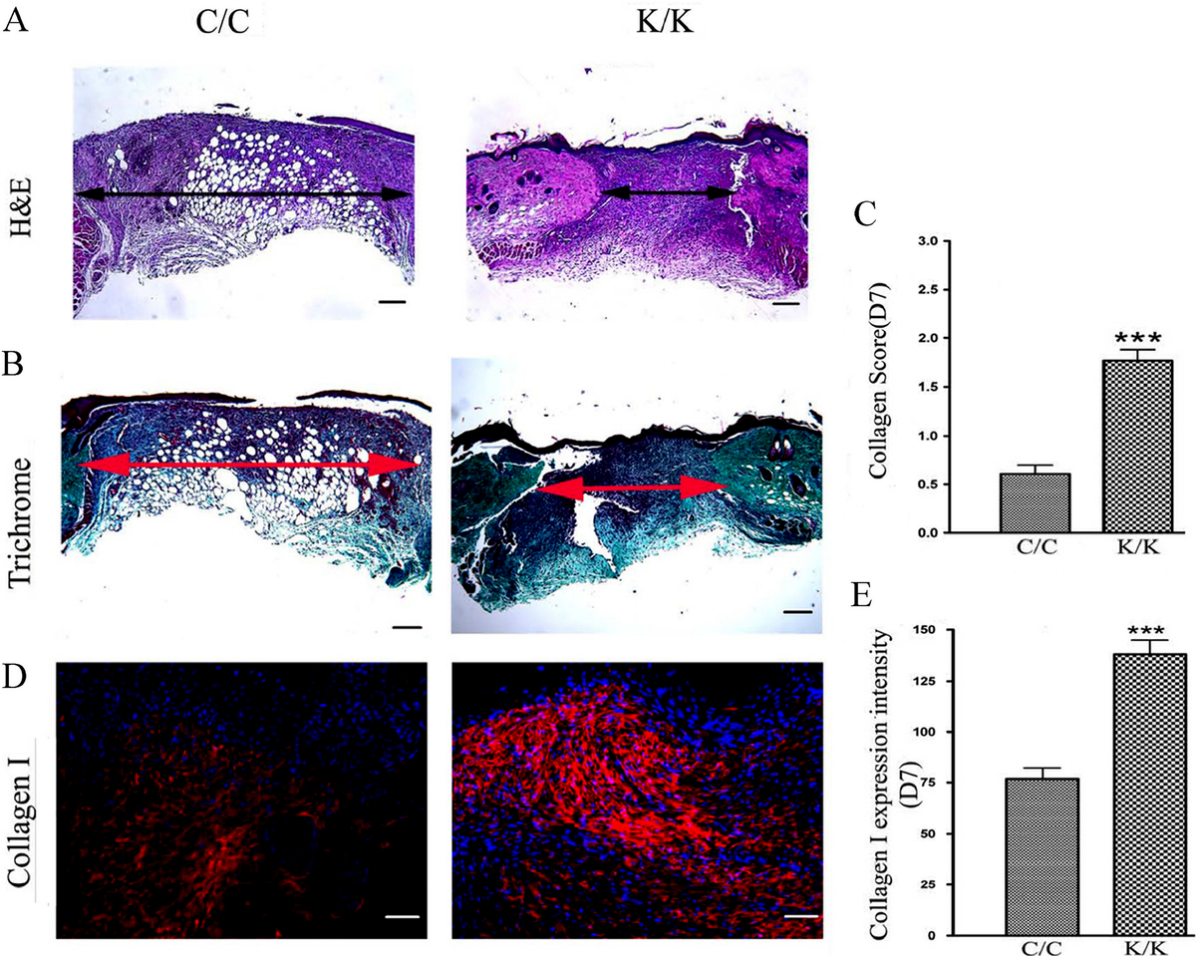
**Histological analysis of PPAR**γ **KO (K/K) mice and WT (C/C) wound tissue**. **(A) **H&E and **(B) **trichrome staining of day 7 wounds (C/C: N = 10; K/K: N = 12, original magnification × 5, bar = 200 μm). The arrow indicates wound width. **(C) **The collagen content in each section was assessed by three blinded observers using the following assessment criteria: 0 signifies no collagen fibers, 1 signifies a few collagen fibers, 2 signifies a moderate amount of collagen fibers, and 3 signifies an excessive amount of collagen fibers. **(D) **Immunofluorescence analysis using an anti-type I collagen antibody. (WT: N = 10; KO: N = 12, original magnification × 20, bar = 50 μm). Red-PPARγ; Blue-DAPI. **(E) **Quantification of type collagen I protein expression in wound tissues at Day 7 after wounding. *** = significant difference between C/C-WT and K/K-KO groups (*P *< 0.001). DAPI, 4',6-diamidino-2-phenylindole; PPARγ, peroxisome proliferator-activated receptor-γ Loss of PPARγ results in enhanced collagen, α-SMA, PCNA and CCN2 expression.

Mice deleted for PPARγ in fibroblasts (K/K) and wild-type littermate control (C/C) mice were generated and genotyped as previously described [9] (Figure 1A). Results were verified using Western blot and indirect immunofluorescence analysis of skin using an anti-PPARγ antibody (Figure 1B, C). To examine whether loss of PPARγ expression in skin fibroblasts affected tissue repair, we subjected mice harboring a fibroblast-specific deletion for PPARγ (K/K) and wild-type littermate control mice (C/C) to the dermal punch model of cutaneous tissue repair. PPARγ knockout mice (K/K) showed significantly increased wound closure three days, five days and seven days post-wounding; however, in both sets of mice, wound closure was essentially complete 10 days post-wounding (Figure 2A, B). The fact that mice harboring a fibroblast-specific deletion for PPARγ (K/K) showed an increased rate of tissue repair was confirmed when skin of mice (seven days post-wounding) were examined histologically using H & E staining (Figure 3A). Thus, in skin, loss of PPARγ increased the kinetics of wound closure

**Figure 4:**
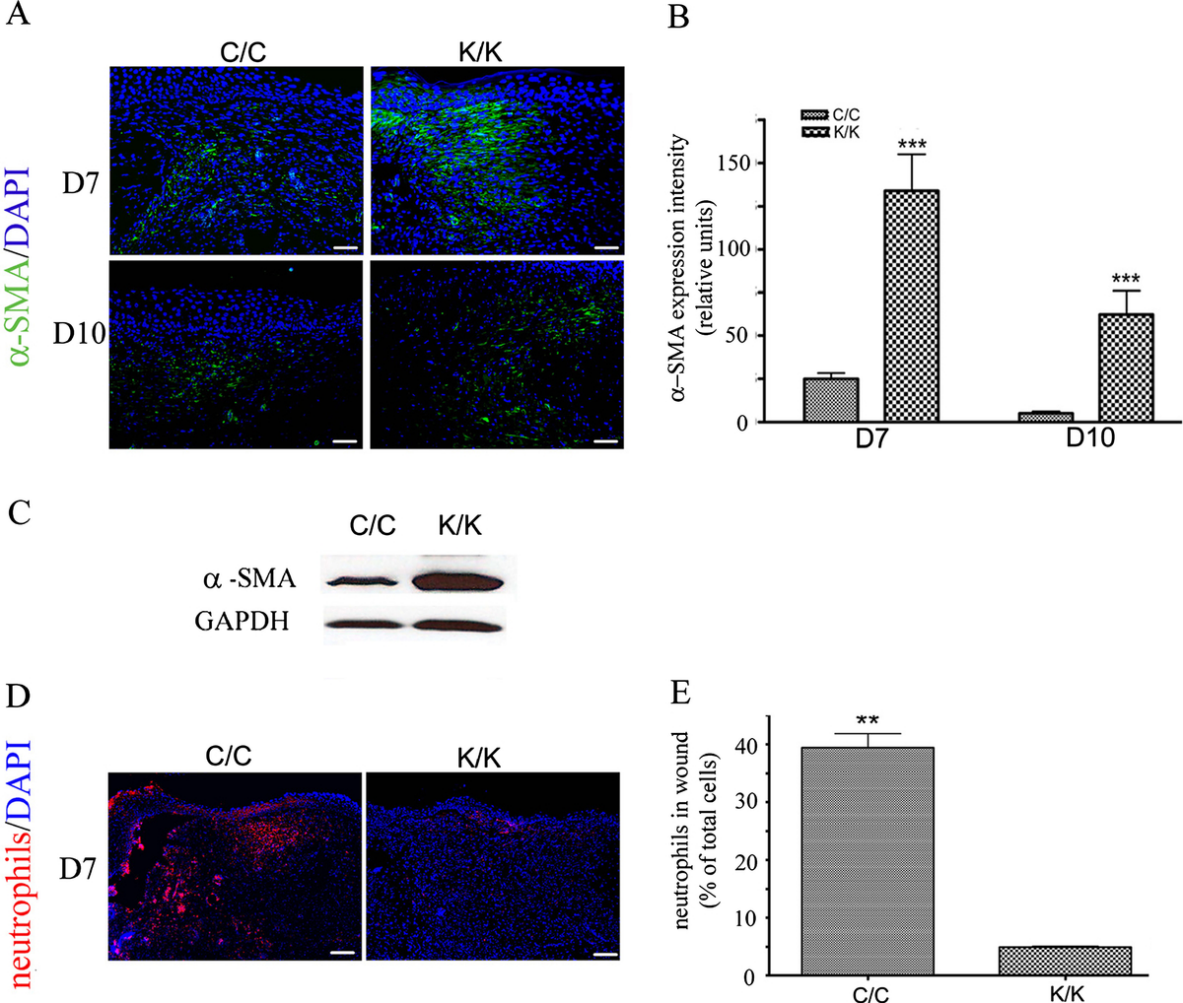
**Alpha-SMA expression is enhanced in PPAR**γ **KO (K/K) wound tissue (day 7 and day 10 post-wounding)**. **(A) **Indirect immunofluorescence analysis of WT (C/C) and PPARγ KO (K/K) mice with an anti-α-SMA antibody (C/C: N = 10; K/K: N = 12, original magnification × 20, bar = 50 μm). Green, α-SMA; Blue, DAPI. **(B) **Quantification of α-SMA expression intensity in wound tissues at day 7 and day 10 after wounding. **(C) **Western blot of α -SMA expression in WT (C/C) and PPARγ KO (K/K) wound tissues at day 7 after wounding. GAPDH = loading control. (**D**) Indirect immunofluorescence analysis of WT (C/C) and PPARγ KO (K/K) mice with an anti-neutrophil antibody (C/C: N = 10; K/K: N = 12, original magnification × 20, bar = 100 μm). Red, neutrophils; Blue, DAPI. **(E) **Quantification of neutrophils in wound tissues at day 7 after wounding. Asterisks indicate a significant difference between C/C and K/K groups (** = *P *< 0.01; *** = *P *< 0.001). DAPI, 4',6-diamidino-2-phenylindole; PPARγ, peroxisome proliferator-activated receptor-γ.

**Figure 5:**
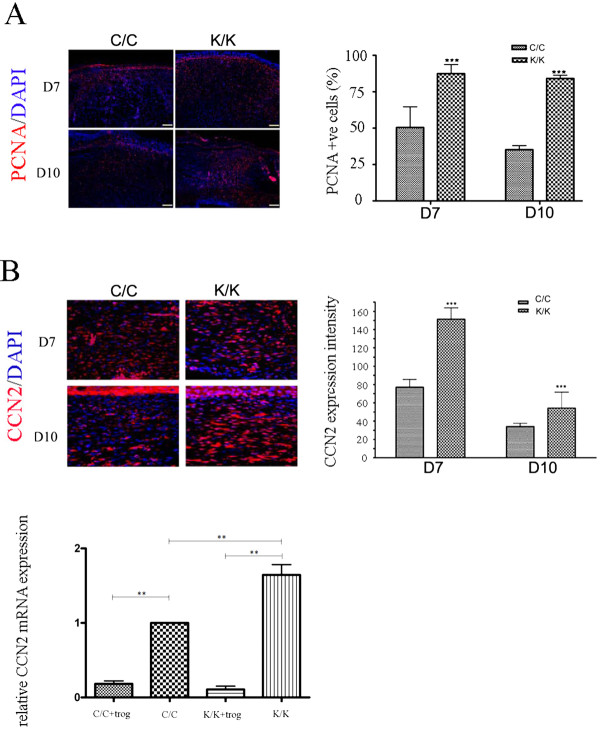
**PCNA and CCN2 expression is enhanced in PPAR**γ **KO (K/K) fibroblasts**. Indirect immunofluorescence analysis of WT (C/C) and PPARγ KO (K/K) mice (day 7 and 10 post-wounding) with **(A) **an anti-PCNA antibody and (**B**) an anti-CCN2 antibody (C/C: N = 10; K/K: N = 12, original magnification × 20, bar = 50 μm). Red, CCN2;Blue, DAPI. *** = indicates significant difference between C/C and K/K groups (*P *< 0.001). **(C) **Real time PCR analysis of WT (C/C) and PPARγ KO (K/K) fibroblasts treated with or without troglitazone (40 μM, 24 hours). Primers detecting CCN2 and 18S mRNAs were used. Expression relative to CCN2 expression in untreated WT (C/C) fibroblasts is shown. Average +/- standard deviation (N = 3) is shown. ** = significant difference between groups (*P *< 0.01). Note that CCN2 mRNA expression was elevated in (K/K) fibroblasts; troglitazone (+trog) reduced CCN2 mRNA expression in both (C/C) and (K/K) fibroblasts indicating that troglitazone operated independent of PPARγ. DAPI, 4',6-diamidino-2-phenylindole; PCNA, proliferating cell nuclear antigen.; PPARγ, peroxisome proliferator-activated receptor-γ Loss of PPARγ results in enhanced Smad3, Akt and ERK phosphorylation.

As loss of PPARγ resulted in an enhanced rate of tissue repair, we assessed whether loss of PPARγ also promoted collagen deposition. Blind histological analysis of trichrome-stained sections showed a greater collagen score in wounded PPARγ (K/K) compared to control (C/C) mice (Figure 3B, C). Assessment of collagen content using an anti-type I collagen antibody (Figure 3D, E) further confirmed that animals deficient in PPARγ possessed elevated collagen levels at day 7 post wounding. We then assessed the effect of loss of PPARγ on myofibroblast induction. Immunohistochemical analysis showed greater expression of a-SMA in day 7 and day 10 wounds of PPARγ knockout (K/K) mice compared to control (C/C) mice (Figure 4A, B). Western blot analysis on protein samples prepared from control and bleomycin-treated animals further confirmed elevated a-SMA production in day 7 wounds of PPAR-deficient (C/C) mice compared to control (K/K) mice (Figure 4C). Fewer neutrophils were found in the wounds of PPAR-deficient (C/C) mice compared to control (K/K) mice (Figure 4D), consistent with the increased rate of wound resolution observed in these mice. Furthermore, PCNA (a marker of cell proliferation) and CCN2 (a marker of tissue repair and fibrosis) expression were also significantly induced in the skin of day 7 and day 10 PPAR -deficient (C/C) mice compared to control (K/K) mice (Figure 5A, B). Similarly, elevated CCN2 mRNA expression was observed in PPAR-deficient fibroblasts (Figure 5C). Intriguingly, troglitazone, a PPARγ agonist, decreased CCN2 mRNA expression in both wild-type and PPARγ-deficient fibroblasts (Figure 5C) suggesting that troglitazone operates through PPAR-independent mechanisms (Figure 5C).

**Figure 6:**
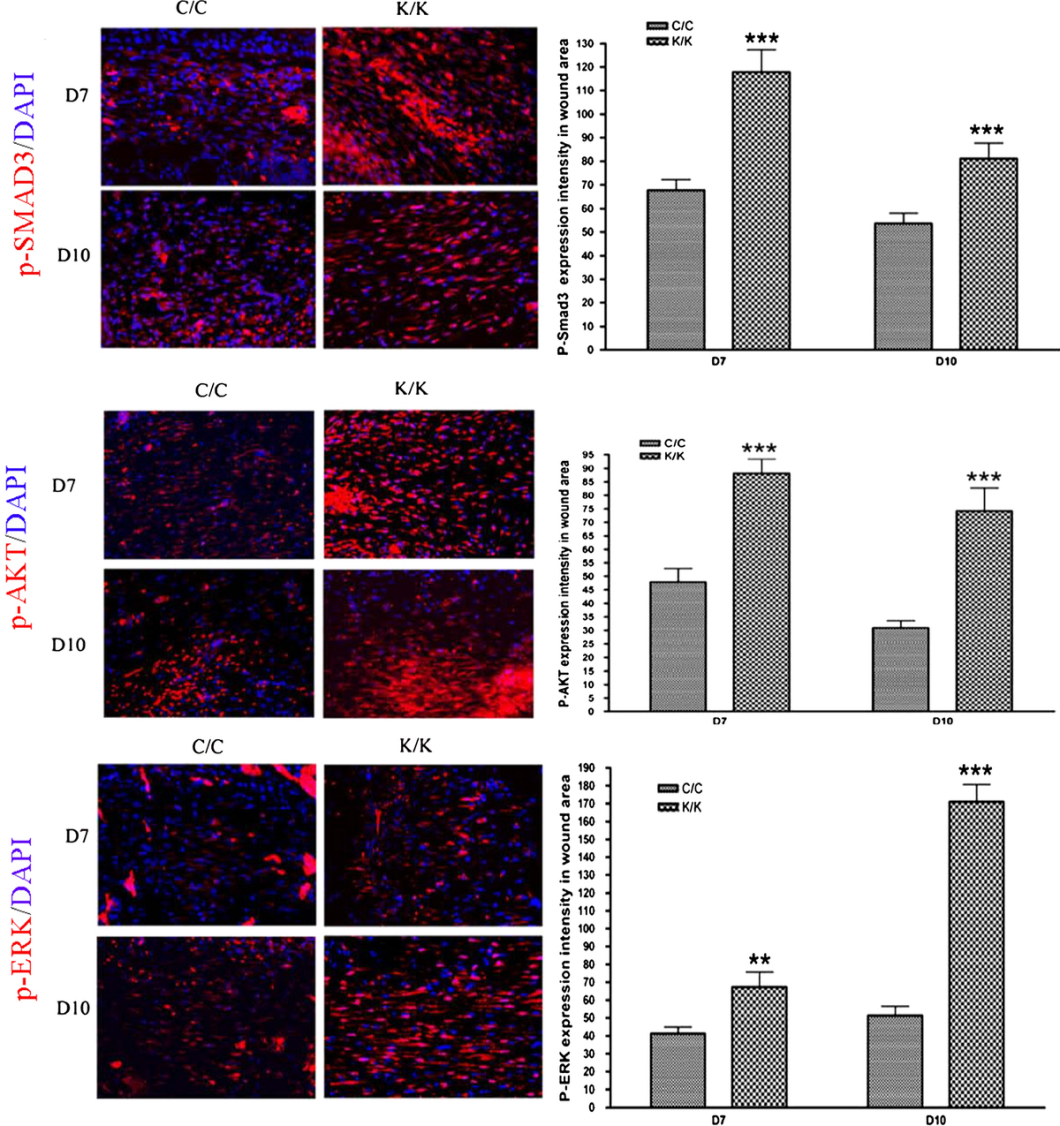
**Elevated p-Akt, p-Smad3 and p-Erk staining in PPAR**γ **KO (K/K) wound tissue (day 7 and day 10 post-wounding)**. Indirect immunofluorescence analysis with anti-, anti-p-Smad3 and anti-p-Erk antibodies, as indicated, in wound tissues day7 and day 10 post-wounding (C/C: N = 10; K/K: N = 12, original magnification × 20, bar = 50 μm). p-Akt, p-Smad3 and p-Erk expression intensity, respectively, are shown. Asterisks indicate a significant difference between C/C and K/K groups (** = *P *< 0.01; *** = *P *< 0.001). PPAR, peroxisome proliferator-activated receptor-γ.

In skin fibroblasts, loss of PPARγ has been shown to result in enhanced phosphorylation of Smad3 in response to bleomycin or TGFβ [9]. Consistent with these data, we showed, using indirect immunofluorescence with an anti-phospho-Smad3, that Smad3 phosphorylation was enhanced seven days post-wounding in PPARγ-deficient animals (Figure 6, p-Smad3). Moreover, enhanced phosphorylation of Akt, a protein that is phosphorylated downstream of TGFβ and has previously been shown to be activated in fibrosis and to be modified by PPARγ [10-14], was also observed in the skin of day 7 and day 10 wounds of PPARγ-deficient animals (Figure 6, p-Akt). Finally, enhanced phosphorylation of ERK, a protein that is also phosphorylated downstream of TGFβ and shown to be modified by PPARγ [14-17], was also observed in the skin of day 7 and day 10 wounds of PPARγ-deficient animals (Figure 6, p-ERK). Collectively, these data indicate that loss of PPARγ expression in skin results in elevated pro-fibrotic signaling.

### Loss of PPARγ does not appreciably affect dermal homeostasis

**Figure 7:**
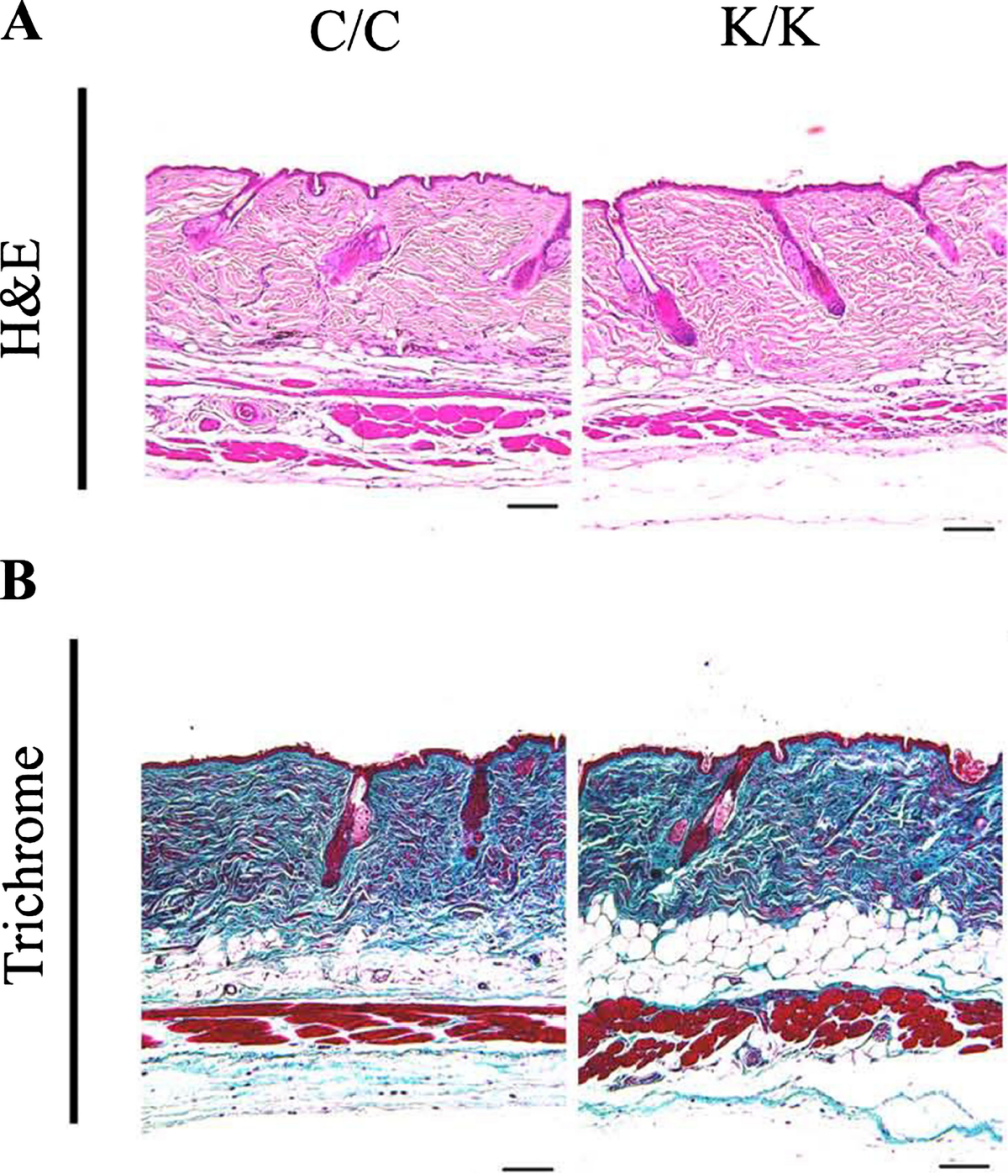
**Loss of PPAR**γ **expression in dermal fibroblasts does not appreciably affect skin homeostasis**. WT (C/C) and PPARγ KO (K/K) skin samples at four months (C/C: N = 5; K/K: N = 6) after tamoxifen injection were examined (original magnification × 10, bar = 100 μm). **(A) **H & E and **(B) **trichrome staining. PPAR, peroxisome proliferator-activated receptor-γ.

*A priori*, we would have expected that, as loss of PPARγ resulted in increased Smad3 and Akt phosphorylation in response to bleomycin or punch wounding [9], this report, loss of PPARγ expression by itself might have been sufficient to result in skin fibrosis. However, PPARγ-deficient skin did not show significant alterations in skin thickness or matrix accumulation even four months after the PPARγ gene was deleted (Figure 7A, B).

Collectively, our results indicate that, *in vivo*, PPARγ normally acts in dermal fibroblasts to suppress fibrogenic responses.

## Discussion

In this study, we tested the effect of loss of PPARγ in dermal fibroblasts on cutaneous tissue repair *in vivo*. PPARγ-deficient mice showed enhanced responsiveness to tissue injury, as shown by increased wound closure on days three, five, seven and ten post-wounding, increased collagen production, the appearance of α-SMA-expressing myofibroblasts, elevated CCN2 expression and enhanced Smad3/Akt phosphorylation. These results are consistent with previous observations that PPARγ-deficient fibroblasts showed enhanced sensitivity to TGFβ1 and that PPARγ-deficient mice showed increased susceptibility to bleomycin-induced skin fibrosis [8,9]. These data also agree with prior studies showing that, in fibroblasts, PPARγ agonists block TGFβ-induced α-SMA and collagen expression [6]. Moreover, we showed that, although CCN2 mRNA was elevated in PPARγ-knockout fibroblasts, troglitazone suppressed CCN2 mRNA in both wild-type and PPARγ-knockout fibroblasts. Although it is possible that these PPARγ agonists were active due to the small residual expression of PPARγ in PPARγ knockout fibroblasts, these data do not exclude the notion that thiazolidinediones such as troglitazone have potent 'off-target' effects independent of PPARγ itself [18]. Nonetheless, these data collectively suggest that PPARγ normally suppresses fibrogenic responses *in vivo *and also support the notion that developing novel classes of direct PPARγ agonists/antagonists is warranted.

We found that long-term (up to four months) deletion of PPARγ did not appreciably affect dermal homeostasis. This result is consistent with our previous data showing that mice deficient in PPARγ expression in fibroblasts (approximately six weeks post-deletion of PPARγ), although possessing enhanced susceptibility to bleomycin-induced skin fibrosis, possessed no detectable alterations in skin structure [9]. PPARγ is known to upregulate the tumor suppressor PTEN; loss of PTEN expression by dermal fibroblasts results in skin fibrosis due to an increase in Akt phosphorylation [13,18]. Intriguingly, we found that PTEN expression was increased and not decreased in the dermis of PPARγ knockout mice (data not shown). These observations suggest that PPARγ knockout mice may upregulate PTEN expression to compensate for the loss of PPARγ expression by dermal fibroblasts and provide a plausible explanation why dermal homeostasis was not appreciably altered in PPARγ knockout mice and injury, whether caused by bleomycin or by dermal punch wounding, is required to elicit a phenotype in PPARγ knockout animals.

## Conclusion

In summary, our studies examining the involvement in PPARγ in skin indicate that PPARγ normally acts in fibroblasts to retard tissue repair. These results suggest that direct PPARγ agonists and antagonists might be used to control the tissue repair program, for example, by suppressing scarring or by promoting the closure of chronic wounds.

## Methods

### Generation of PPARγ conditional knockout mice

Mice, hemizygous for an allele expressing tamoxifen-inducible Cre-recombinase (under the control of a fibroblast-specific regulatory sequence from the proa2(I) collagen gene [19]) and homozygous for a loxP-PPARγ allele, were generated as described previously [9]. Mice deleted (K/K) or not (C/C) for PPARγ in fibroblasts were generated by treating three-week-old mice each day for five days with tamoxifen (4-hydroxitamoxifen, Sigma, St. Louis, MO, USA) in corn oil (0.1 ml of 10 mg/ml) or corn oil. Deletion of PPARγ was verified by PCR genotyping (Jackson Laboratories, Bar Harbor, ME, USA).γ All animal protocols were approved by the regulatory authority of the appropriate experimental animal committee.

### Cell culture, immunofluorescence and Western analysis

Dermal fibroblasts, isolated from explants (four- to six-week-old animals) as described, were subjected to indirect immunofluorescence analysis followed by an appropriate secondary antibody (Jackson Immunoresearch, West Grove, PA, USA) as described [9]. Photography (Zeiss Axiphot) was performed using a digital camera (Empix, Mississauga, ON, Canada). For some assays, cells were lysed in 2% SDS, proteins quantified (Fisher, Nepean, ON, Canada) and subjected to Western blot analysis [9]. Antibodies used were: anti-a-SMA (Sigma,1:3000), anti-β-actin (1:5000, Sigma) and anti- PPARγ (Santa Cruz, Santa Cruz, CA, USA,1:500).γ Human dermal fibroblasts were purchased (American Type Culture Collection, Manassas, VA, USA). Cells were grown in (D)MEM, 10% fetal bovine serum (Life Technologies, Burlington, ON, Canada).

### Assessment of collagen content

To assess the effects of PPARγ deletion on collagen synthesis, trichrome collagen stain was used. Collagen content in each section was assessed by three blinded observers using the following assessment criteria: 0 signifies: no collagen fibers; 1 signifies: few collagen fibers; 2 signifies: moderate amount of collagen fibers; 3 signifies: excessive amount of collagen fibers.

### Immunohistochemistry

Non-specific immunoglobulin G (IgG) binding was blocked by incubating sections with BSA (0.1%) in PBS for 1 hour and then incubated with primary antibody in a humidified chamber and left overnight at 4°C. Next, sections were washed and incubated with a secondary antibody for 1 hour. Primary antibodies used were: PPARγ (Santa Cruz, Rabbit, sc-7196, 1:500) PTEN (Cell Signaling, Pickering, ON, Canada, #9559, Rabbit, 1:500) CCN2 (Abcam, Cambridge, MA, USA, ab6992, Rabbit, 1:250), α-SMA (Sigma, A5228, mouse, 1:2000); ColIa2 (Santa Cruz, sc-28654, rabbit, 1:500), PCNA: (Abcam, ab2426-1, rabbit, 1:500); p-SMAD3: (Abcam, rabbit, ab52903, 1:200); p-AKT (Rockland Immunochemicals, Gilbertsville, PA, USA, 200-301-268, mouse), (1:500); p-ERK (Cell Signaling, #9101, rabbit, 1:500) and neutrophil marker (Santa Cruz, sc-59338, rat,1:100). Secondary antibodies (Jackson Immunoresearch, 1:400) were: Dylight 488 conjugated donkey anti-mouse (711-485-150); Dylight 594 conjugated donkey anti-rabbit (711-485-152); Dylight 594 conjugated donkey anti-mouse (715-515-150) and Dylight 594 conjugated donkey anti-rat (712-516-150).

### Real-time PCR

Real-time PCR to detect the expression of target genes was performed essentially as previously described [20,21]. Cells were cultured until 50% confluence and treated for 24 hours with dimethyl sulfoxide (DMSO) or troglitazone (40 μM, EMD Biosciences, Billerica, MA, USA) and total RNA was isolated (RNeasy; QIAGEN, Toronto, ON, Canada). Total RNA (25 ng) was reverse transcribed, amplified using Taq-Man Assays-on-Demand in the presence One-Step MasterMix and detected using the ABI Prism 7900 HT sequence detector (Life Technologies). Triplicate samples were run, and expression values for CCN2 were standardized to values obtained with control 18S primers using the ΔΔCt method.

### Statistics

Statistical analysis was done using one way analysis of variance (ANOVA) and Tukey's post-hoc test on Graphpad Prism 4 software.

## Abbreviations

bp: Base pair; BSA: Bovine serum albumin; DAPI: 4',6-diamidino-2-phenylindole; (D)MEM: (Dulbecco's) modified Eagle's medium; H & E: Hematoxylin and eosin; IgG: Immunoglobulin G; PCNA: Proliferating cell nuclear antigen; PCR: Polymerase chain reaction; PPAR: Peroxisome proliferator-activated receptorγ; RXR: Retinoid X receptor; α-SMA: α-smooth muscle actin; TGF-β: Transforming growth factor-β.

## Competing interests

The authors declare that they have no competing interests.

## Authors' contributions

WS conducted the *in vivo *studies and tissue analysis. KT and JS carried out the cell culture and real time PCR analysis. MB participated in design of the study. AL conceived of the study, and participated in its design and coordination. WS, KT, MB and AL wrote the paper. All authors read and approved the final manuscript.

## References

[B1] Tomasek JJ Gabbiani G Hinz B Brown RA Myofibroblasts and mechano-regulation of connective tissue remodelling Nat Rev Mol Cell Biol 2003 3 349 363 11988769 10.1038/nrm809

[B2] Elliott CG Hamilton DW Deconstructing fibrosis research: do pro-fibrotic signals point the way for chronic dermal wound regeneration? J Cell Commun Signal 2011 5 301 315 10.1007/s12079-011-0131-5 21503732 PMC3245760

[B3] Shi-wen X Eastwood M Stratton RJ Denton CP Leask A Abraham DJ Rosiglitazone alleviates the persistent fibrotic phenotype of lesional skin scleroderma fibroblasts Rheumatology (Oxford) 2010 49 259 263 10.1093/rheumatology/kep371 20007285

[B4] Plutzky J The PPAR-RXR transcriptional complex in the vasculature: energy in the balance Circ Res 2011 108 1002 1016 10.1161/CIRCRESAHA.110.226860 21493923

[B5] Wei J Bhattacharyya S Varga J Peroxisome proliferator-activated receptor γ innate protection from excessive fibrogenesis and potential therapeutic target in systemic sclerosis Curr Opin Rheumatol 2010 22 671 676 10.1097/BOR.0b013e32833de1a7 20693905 PMC4536822

[B6] Burgess HA Daugherty LE Thatcher TH Lakatos HF Ray DM Redonnet M Phipps RP Sime PJ PPARgamma agonists inhibit TGF-beta induced pulmonary myofibroblast differentiation and collagen production: implications for therapy of lung fibrosis Am J Physiol Lung Cell Mol Physiol 2005 288 L1146 L1153 10.1152/ajplung.00383.2004 15734787

[B7] Wu M Melichian DS Chang E Warner-Blankenship M Ghosh AK Varga J Rosiglitazone abrogates bleomycin-induced scleroderma and blocks profibrotic responses through peroxisome proliferator-activated receptor-gamma Am J Pathol 2009 174 519 533 10.2353/ajpath.2009.080574 19147827 PMC2630560

[B8] Ghosh AK Wei J Wu M Varga J Constitutive Smad signaling and Smad-dependent collagen gene expression in mouse embryonic fibroblasts lacking peroxisome proliferator-activated receptor-gamma Biochem Biophys Res Commun 2008 374 231 236 10.1016/j.bbrc.2008.07.014 18627765 PMC3157939

[B9] Kapoor M McCann M Liu S Huh K Denton CP Abraham DJ Leask A Loss of PPARγ in mouse fibroblasts results in increased susceptibility to bleomycin-induced skin fibrosis Arthritis Rheum 2009 60 2822 2829 10.1002/art.24761 19714649

[B10] Shi-wen X Stanton LA Kennedy L Pala D Chen Y Howat SL Renzoni EA Carter DE Bou-Gharios G Stratton RJ Pearson JD Beier F Lyons KM Black CM Abraham DJ Leask A CCN2 is necessary for adhesive responses to transforming growth factor-beta1 in embryonic fibroblasts J Biol Chem 2006 281 10715 10726 10.1074/jbc.M511343200 16484225

[B11] Kulkarni AA Thatcher TH Olsen KC Maggirwar SB Phipps RP Sime PJ PPAR-γ ligands repress TGFβ-induced myofibroblast differentiation by targeting the PI3K/Akt pathway: implications for therapy of fibrosis PLoS One 2011 6 e15909 10.1371/journal.pone.0015909 21253589 PMC3017065

[B12] Xu SW Liu S Eastwood M Sonnylal S Denton CP Abraham DJ Leask A Rac inhibition reverses the phenotype of fibrotic fibroblasts PLoS One 2009 4 e7438 10.1371/journal.pone.0007438 19823586 PMC2757676

[B13] Parapuram SK Shi-wen X Elliott C Welch ID Jones H Baron M Denton CP Abraham DJ Leask A Loss of PTEN expression by dermal fibroblasts causes skin fibrosis J Invest Dermatol 2011 131 1996 2003 10.1038/jid.2011.156 21654839

[B14] Huang W András IE Rha GB Hennig B Toborek M PPARα and PPARγ protect against HIV-1-induced MMP-9 overexpression via caveolae-associated ERK and Akt signaling FASEB J 2011 25 3979 3988 10.1096/fj.11-188607 21840940 PMC3205841

[B15] Leask A Holmes A Black CM Abraham DJ Connective tissue growth factor gene regulation. Requirements for its induction by transforming growth factor-beta 2 in fibroblasts J Biol Chem 2003 278 13008 13015 10.1074/jbc.M210366200 12571253

[B16] Zhang M Fraser D Phillips A ERK, p38, and Smad signaling pathways differentially regulate transforming growth factor-beta1 autoinduction in proximal tubular epithelial cells Am J Pathol 2006 169 1282 1293 10.2353/ajpath.2006.050921 17003485 PMC1698849

[B17] Van Beek JP Kennedy L Rockel JS Bernier SM Leask A The induction of CCN2 by TGFbeta1 involves Ets-1 Arthritis Res Ther 2006 8 R36 10.1186/ar1890 16469114 PMC1526589

[B18] Zhang W Wu N Li Z Wang L Jin J Zha XL PPARgamma activator rosiglitazone inhibits cell migration via upregulation of PTEN in human hepatocarcinoma cell line BEL-7404 Cancer Biol Ther 2006 5 1008 1014 10.4161/cbt.5.8.2887 16775433

[B19] De Val S Ponticos M Antoniv TT Wells DJ Abraham D Partridge T Bou-Gharios G Identification of the key regions within the mouse pro-alpha 2(I) collagen gene far-upstream enhancer J Biol Chem 2002 277 9286 9292 10.1074/jbc.M111040200 11756428

[B20] Fajardo OA Thompson K Parapuram SK Liu S Leask A Mithramycin reduces expression of fibro-proliferative mRNAs in human gingival fibroblasts Cell Prolif 2011 44 166 173 10.1111/j.1365-2184.2011.00738.x 21401758 PMC6495922

[B21] Thompson K Hamilton DW Leask A ALK5 inhibition blocks TGFβ-induced CCN2 expression in gingival fibroblasts J Dent Res 2010 89 1450 1454 10.1177/0022034510379020 20924066

